# Spinal meningiomas, from biology to management - A literature review

**DOI:** 10.3389/fonc.2022.1084404

**Published:** 2023-01-13

**Authors:** Nicolas Serratrice, Imène Lameche, Christian Attieh, Moussa A Chalah, Joe Faddoul, Bilal Tarabay, Rabih Bou-Nassif, Youssef Ali, Joseph G Mattar, François Nataf, Samar S Ayache, Georges N Abi Lahoud

**Affiliations:** ^1^ Institut de la Colonne Vertébrale et des Neurosciences (ICVNS), Centre Médico-Chirurgical Bizet, Paris, France; ^2^ EA 4391, Excitabilité Nerveuse et Thérapeutique, Faculté de Santé, Université Paris Est, Créteil, France; ^3^ Gilbert and Rose-Marie Chagoury School of Medicine, Lebanese American University, Byblos, Lebanon; ^4^ Service de Neurochirurgie, Centre Hospitalier de la Côte Basque, Bayonne, France; ^5^ Department of Neurosurgery, Memorial Sloan Kettering Cancer Center, New York, NY, United States; ^6^ Institut de Chirurgie Osseuse et de Neurochirurgie, Médipole-Montagard, Avignon, France; ^7^ Service de Neurochirurgie, Hôpital Lariboisière, Paris, France; ^8^ Service de Physiologie-Explorations Fonctionnelles, DMU FIxIT, Hôpital Henri Mondor, Créteil, France

**Keywords:** gross total resection (GTR), meningiomas, spinal meningiomas, stereotactic body radiation therapy (SBRT), minimally invasive (MIS), microsurgery (MS), ultrasonic dissection, central nervous system

## Abstract

Meningiomas arise from arachnoidal cap cells of the meninges, constituting the most common type of central nervous system tumors, and are considered benign tumors in most cases. Their incidence increases with age, and they mainly affect females, constituting 25-46% of primary spinal tumors. Spinal meningiomas could be detected incidentally or be unraveled by various neurological symptoms (*e.g.*, back pain, sphincter dysfunction, sensorimotor deficits). The gold standard diagnostic modality for spinal meningiomas is Magnetic resonance imaging (MRI) which permits their classification into four categories based on their radiological appearance. According to the World Health Organization (WHO) classification, the majority of spinal meningiomas are grade 1. Nevertheless, they can be of higher grade (grades 2 and 3) with atypical or malignant histology and a more aggressive course. To date, surgery is the best treatment where the big majority of meningiomas can be cured. Advances in surgical techniques (ultrasonic dissection, microsurgery, intraoperative monitoring) increase the complete resection rate. Operated patients have a satisfactory prognosis, even in those with poor preoperative neurological status. Adjuvant therapy has a growing role in treating spinal meningiomas, mainly in the case of subtotal resection and tumor recurrence. The current paper reviews the fundamental epidemiological and clinical aspects of spinal meningiomas, their histological and genetic characteristics, and their management, including the various surgical novelties and techniques.

## Introduction

Meningiomas are lesions that arise from arachnoidal cap cells, the outer part of the arachnoid layer and villi (1). They usually form dural attachments and are marked by meningothelial hyperplasia (1).

Spinal meningiomas are relatively rare, accounting for approximately 3% of all meningiomas of the central nervous system (CNS) (2) and 25-46% of all primary intraspinal neoplasms (2, 3). 90% of intradural extramedullary spinal tumors are either meningiomas or schwannomas and constitute nearly 25% of primary spinal neoplasia (3).

Spinal meningiomas mainly affect people in their fifth decade of life and are more frequent in women (given their estrogen receptors) (4). In case they occur in young patients, or if they cause multiple lesions, a genetic disorder like neurofibromatosis type 2 (NF2) or aggressive histological subtypes should be suspected (2, 4). Spinal meningiomas most commonly occur in the posterior, posterolateral, or lateral thoracic region, followed by the anterior cervical and lumbosacral regions. The main treatment is surgery, which can be performed in (a) a classical open microsurgical approach in the vast majority of cases, (b) a minimally invasive surgery (MIS), or (c) through an endoscopic intervention (5). The choice of the most appropriate surgical method could be challenging, especially in the case of aggressive meningiomas and in some difficult access tumors (*e.g.*, location anterior to the spinal cord in the thoracic region). For instance, ventral/ventrolateral cervical meningiomas within the upper cervical region, may envelop the vertebral artery (6), and thus require precise presurgical calculations to adopt the safest approach.

The scientific knowledge regarding spinal meningiomas has evolved over the past twenty years. This evolution concerns multiple aspects, including clinical evaluation, molecular and radiological specificities, and microsurgical management techniques. Therefore, the main goal of this paper is to come up with an updated review of spinal meningiomas, with a particular focus on their clinical presentation, biological and imaging aspects, and neurosurgical strategies.

## Selection criteria

A review of the literature was carried out using PubMed/Medline and Scopus databases. The following keywords were used: “spinal meningioma” AND (“biology” OR “molecular” OR “genetic” OR “endocrine” OR “imaging” OR “grading” OR “prognosis” OR “surgery” OR “resection” OR “intraoperative monitoring” OR “adjuvant therapy” OR “recurrence” OR “psychology” OR “anxiety” AND “depression”). Original papers published in English and French were included in the analysis. The references of the selected papers were manually scanned in order to identify any additional references. To note, papers on intracranial meningiomas were also scanned and relevant papers were included since some research on spinal meningiomas stem from studies carried out on intracranial meningiomas.

## Clinical features

**Figure 1:**
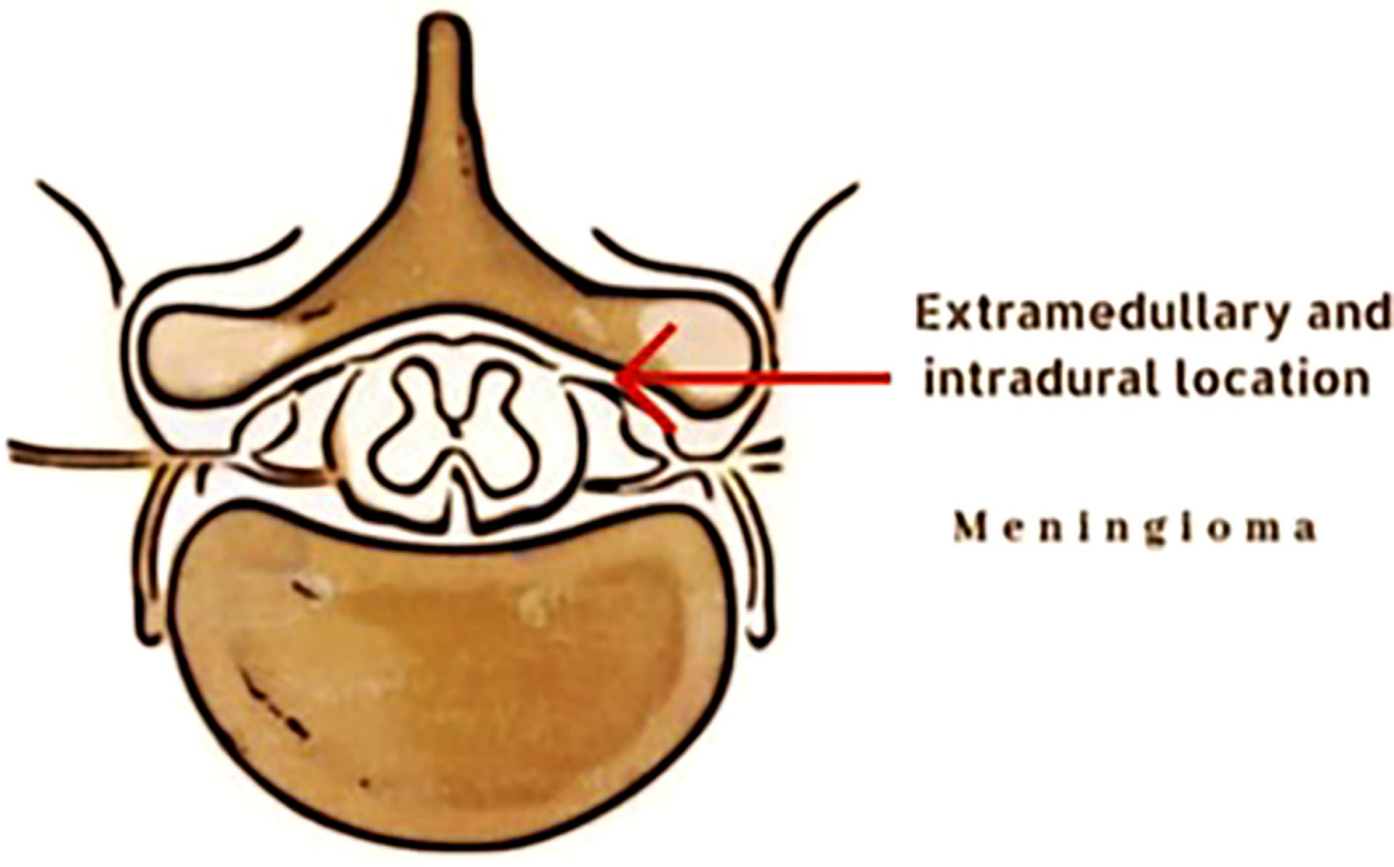
Illustration of extramedullary and intradural location of a spinal meningioma.

Spinal meningiomas are typically solitary, well-circumcised, slow-growing, intradural extramedullary tumors (**Figure 1**) (7) that usually respect the surrounding normal tissue (1). Hence, they are often perceived as noninvasive. However, they could be aggressive in some cases by seeding other parts of the CNS or the surgical site.

As stated above, meningiomas are more frequently seen at the thoracic level and tend to expand slowly without any clinical manifestation initially. Around 9% of cases are asymptomatic and report no complaints (8). At an advanced stage, meningiomas compromise spinal elements, resulting in various neurological symptoms and signs, such as pain, motor, and sensory disturbances, gait abnormality, and sphincter dysfunction. Complaints can vary from one person to another depending on the size of the meningioma and the exact site of the spinal compression (8). Clinical manifestations depend also on the initial size of the spinal canal as large constitutional ones can tolerate the development of large sized tumors while remaining asymptomatic.

The most frequent presentation is pain, ranging from 42% in some case series to as high as 87% in other reports (8, 9). Pain is more frequently described as local or radicular. Apart from pain, patients often report sensory and motor symptoms. Sensory manifestations can have many aspects, such as aching, burning, tingling, numbness, hypoesthesia, paresthesia, and anesthesia. These symptoms can be present in 16% to 84% of cases (4, 10). However, the most alarming sign for patients is motor deficit. It can start as a slight weakness and evolves later into a complete motor deficit. Motor symptoms are present in 33% to 93% of patients with spinal meningiomas (2, 4). Moreover, some patients could complain of gait and balance disturbances in 47% to 93% of cases (8, 11). Finally, sphincter dysfunction is reported in some series but less frequently than other symptoms. Sphincter dysfunction can be seen in 6 to 60% of patients seeking medical opinion (8, 11). A summary of the clinical manifestations is depicted in **Table 1**.

**Table 1:** Summary of clinical manifestations in spinal meningiomas.

Authors	Patients	Meningiomas location	Symptoms/Signs
Levy et al., 1982 (12)	n=97 (mean age 53, 80% females)	Cervical (n=17) Thoracic (n=73) Lumbar (n=7)	Back and/or limb pain 72% Motor deficit 66% Sensory symptoms +/- signs 32% Bowel and/or bladder dysfunction 40% Gait disturbances NP
Solero et al., 1989(13)	n=174 (mean age 56, 82% females)	Cervical (n=26) Thoracic (n=144) Lumbar (n=4)	Back and/or limb pain 53% Motor deficit 93% Sensory symptoms +/- Signs 61% Bowel and/or bladder dysfunction 50% Gait disturbances NP
Gezen et al., 2000(14)	n=36 (mean age 49, 75% females)	Cervical (n=8) Thoracic (n=20) Lumbar (n=8)	Back and/or limb pain 83% Motor deficit 83% Sensory symptoms +/- signs 50% Bowel and/or bladder dysfunction 36% Gait disturbances NP
Cohen-Gadol et al., 2003(15)	n=40 (mean age 34.5, 87.5% females)	Cervical (n=16) Thoracic (n=23) Lumbar (n=2)	Back and/or limb pain 45% Motor deficit 40% Sensory symptoms +/- signs 80% Bowel and/or bladder dysfunction 40% and 13% respectively Gait disturbances 68%
Cohen-Gadol et al., 2003(15)	n=40 (mean age 67.1, 82.5% female)	Cervical (n=8) Thoracic (n=32) Lumbar (n=0)	Sensory symptoms +/- signs 87% Bowel and/or bladder dysfunction 40% and 8% respectively Gait disturbances 80%
Sandalcioglu et al., 2008(4)	n=131 (mean age 69, 87% females)	Cervical (n=21) Cervicothoracic (n=7) Thoracic (n=95) Thoracolumbar (n=6) Lumbar (n=2)	Back and/or limb pain 47% Motor deficit and sensory symptoms +/- signs 84% Gait disturbances 83%
Engelhard et al., 2010(8)	n=430 (mean age 49.3, 56.7% females)		Back and/or limb pain 42% Motor deficit 64% Sensory symptoms +/- signs 50% Bowel and/or bladder dysfunction 16% and 6% respectively Gait disturbances 47%
Postalci et al., 2011(9)	n=46 (mean age 52, 72% females)	Cervical (n=4) Thoracic (n=39) Lumbar (n=3)	Back and/or limb pain 87% Motor deficit 78% Bowel and/or bladder dysfunction 9% Asymptomatic 9% Gait disturbances NP
Riad et al., 2013(11)	n=15 (mean age 67.6, 86.7% females)	Cervical (n=2) Thoracic (n=11) Lumbar (n=2)	Back and/or limb pain 60% Motor deficit 80% Sensory symptoms +/- signs 80% Bowel and/or bladder dysfunction 60% Gait disturbances 93%
Zhang et al., 2020(10)	n=84 (mean age NP, 63.1% females)	Cervical (n=10) Cervicothoracic (n=1) Thoracic (n=4) Thoracolumbar (n=13) Lumbar (n=56)	Back and/or limb pain 75% Motor deficit 33% Sensory symptoms +/- signs 16% Bowel and/or bladder dysfunction 11% Gait disturbances NP

N, number; NP, Not Provided; age is expressed in years.

## Pathology

**Figure 2:**
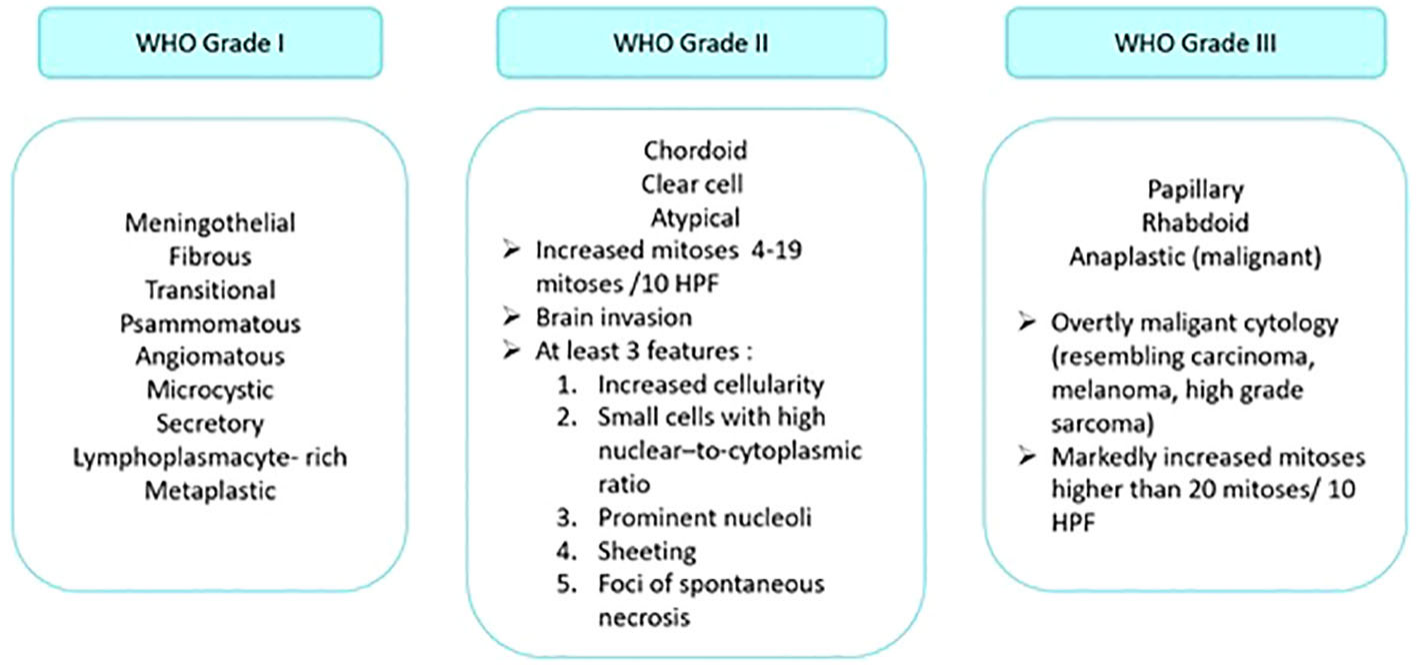
Grading of meningiomas according to the 2021 WHO classification system. HPF: high power field.

Many histological subtypes of meningiomas have been described, among which meningothelial, fibroblastic, and transitional meningiomas are the most frequent ones. The histological analysis of meningiomas defines the histological type and grade of the tumor according to the 2021 World Health Organization (WHO) classification, as illustrated in **Figure 2** (16). It classifies meningiomas into three grades, where each grade correlates with different potential for growth, metastatic spread, recurrence, and prognosis.

Many histologic subtypes are observed in both intracranial and spinal meningiomas. Meningothelial, metaplastic, psammomatous, transitional, atypical, and clear cell subtypes are the most common subtypes of intracranial meningiomas. As for spinal meningiomas, the psammomatous, meningothelial and transitional subtypes distinguish them, and they have a lower risk of recurrence than the intracranial ones for reasons yet to be determined.

The WHO divides meningiomas into three grades from 1 to 3 (benign, atypical and malignant, respectively) based on their malignancy degree (17). In the case of “mixed” tumors, the diagnosis of the dominant histological type (*i.e.*, the type forming more than 50% of the tumor) is retained. However, the presence of a minority contingent with more aggressive potential should be reported.

## Genetic alterations

Advances in molecular biology have greatly improved the understanding of the various mechanisms at the origin of meningiomas development. Molecular profiles of spinal meningiomas are similar to their intracranial counterparts (18). These alterations account for 3% of reported cases only. Chromosomal instability is a widespread molecular alteration that characterizes recurrent or poor prognosis meningiomas. Accumulation of cytogenetic aberrations is associated with higher-grade meningiomas and a higher risk of recurrence, which explains why high-grade meningiomas have more altered cytogenetic profiles than benign meningiomas (19). **Table 2** summarizes the different gene mutations implicated in meningiomas and their prognosis.

**Table 2:** Main molecular alteration and their prognosis in meningiomas.

Authors	Genes	Products	Gene alterations	Prognosis
Mota et al., 2020 (20)	NF2	Merlin	Downregulation Several mutations	Early event in tumorigenesis
Weber et al., 1997 (19), Goutagny et al., 2010(21)	CDKN2A/p14ARF	P14	Downregulation Hypermethylation	Associated with high-grade tumors
Weber et al., 1997 (19), Goutagny et al., 2010(21)	ALPL	Alkaline phosphatase	Downregulation	Associated with high-grade tumors and recurrence
Lee & Lee, 2020 (22)	PI3K	Catalytic subunit of kinase, PI3K	Upregulation	Associated with tumorigenesis of non-NF2 meningiomas
Lee & Lee, 2020 (22)	AKT1	Serine/threonine-protein kinase	Upregulation E17K mutation	Associated with tumorigenesis of non-NF2 meningiomas
Lee & Lee, 2020 (22)	TRAF7	TNF receptor-associated factor 7	Several mutations	Associated with tumorigenesis of non-NF2 meningiomas
Lee & Lee, 2020 (22)	KLF4	Kruppel-like factor 4	Upregulation K409Q mutation	Associated with tumorigenesis of non-NF2 and secretory meningiomas
Lee & Lee, 2020 (22)	SMO	Smoothened, G protein-coupled receptor	Upregulation Several mutations	Associated with tumorigenesis of non-NF2 meningiomas
Tauziede-Espariat et al., 2018 (15)	SMARCE1	Subunit of the SWI/SNF complex	Downregulation Several mutations	Associated with high-grade tumors
Tauziede-Espariat et al., 2018 (15)	SMARCB1	Subunit of the SWI/SNF complex	Several mutations	Associated with high-grade tumors
Barresi et al., 2012 (23)	MMP9	Matrix metalloproteinase-9	Upregulation	Associated with tumorigenesis and edema

AKT1, Alpha serine/threonine-protein kinase 1; ALPL, Alkaline phosphatase; CDKN2A, Cyclin-dependent kinase inhibitor 2A; KLF4, Kruppel-like factor 4; MMP9, Matrix metallopeptidase 9; NF2, Neurofibromatosis type 2; PI3K, Phosphoinositide 3-kinases; SNF, Sucrose Non-Fermentable; SWI, SWItch; SMARCB1, SWI/SNF related, matrix associated, actin dependent regulator of chromatin, subfamily B, member 1; SMARCE1, SWI/SNF related, matrix associated, actin dependent regulator of chromatin, subfamily E, member 1; SMO, Smoothened; TNF, Tumor necrosis factor; TRAF7, TNF receptor-associated factor 7.

Among the multiple mechanisms of oncogenesis, the increased cell proliferation was considered as the most important one. In brain tumors, the typical immunohistochemically marker Ki-67/MIB-1 for cell proliferation is more predictive of survival than the expression of proliferating cell nuclear antigens p53 and PCNA. In meningiomas, a high expression level of Ki-67 is directly associated with significant worse prognostic, especially with Ki-67 index higher than 4% (24). A close follow-up is recommended in this population.

### Chromosomal abnormalities

Among the chromosomal abnormalities found to be related to spinal meningiomas, chromosomal 22q deletion seems to be the most important one (25). In addition, one study on sixteen patients with spinal meningiomas showed an allelic loss of the 1p chromosomal arm - involving several genes such as the ALPL (Alkaline phosphatase) gene - and a homozygous loss of 9p that results in inactivation of tumor suppressor genes such as the CDKN2A (Cyclin-dependent kinase inhibitor 2A) (19, 21). Other chromosomal abnormalities have also been described, such as the loss of 10q and the gain of 5p and 17q (26).

### NF2-mutated meningiomas

In Schwann cells, the NF2 gene on chromosome 22q12.2 leads to the production of merlin, a protein also known as schwannomine, that provides myelin insulation for nerves. Merlin is also involved in the regulation of several key signaling pathways implied in cytoskeletal remodeling and cell motility. In addition, this protein is a tumor suppressor that prevents cell proliferation (20). The loss of merlin expression is characteristic of all NF2-associated meningiomas and nearly half of sporadic cases. Mutations in the NF2 gene produce an abnormally shortened protein altering its functional condition (20). NF2 mutation is also known to activate several oncogenic signaling pathways such as PI3K/AKT1 (20).

### NF2-non-mutated meningiomas

Approximately 40% of sporadic meningiomas are independent of NF2 inactivation and are linked to other mutations discovered in high-throughput sequencing studies of large cohorts of meningiomas (27). For grade I meningiomas, mutations in AKT1 (v-akt murine thymoma viral oncogene homolog 1, leading to activation of the PI3K pathway), TRAF7 (Tumor necrosis factor receptor-associated factor 7, encoding the pro-apoptotic E3 ubiquitin ligase), KLF4 (Krupple-like factor 4, a pluripotency-inducing transcription factor) and SMO (Smoothened, frizzled family receptor, leading to activation of the Hedgehog pathway) have been identified and appear to be mutually exclusive of NF2 alterations (22). In addition, mutations in the SMARCE1 gene (SWI/SNF related, matrix associated, actin dependent regulator of chromatin, subfamily E, member 1) have been reported in clear cell meningioma, and mutations in the SMARCB1 gene (SWI/SNF related, matrix associated, actin dependent regulator of chromatin, subfamily B, member 1) are involved in multiple meningiomas. These mutations are further associated with tumor location and histological subtype (Tauziede-Espariat et al., 2017). In spinal schwannoma, one study reported a mutation in the large tumor suppressor kinase 1 gene (LATS1), a downstream mediator of NF2, but the clinical relevance of these alterations remains unknown in spinal meningiomas (28).

### Other additional mutations

Comparative microarray analysis between spinal and intracranial meningiomas confirmed that spinal meningiomas are associated to a higher rate of chromosome 22 deletion (23). Moreover, 35 genes out of 1555 reported were more highly expressed in spinal than in intracranial meningiomas (23). Barresi et al. reported that spinal meningiomas showed an increased expression of the matrix metalloproteinase family, a group of proteins also involved in cell growth and invasion (23).

## Meningiomas and sex hormones

Multiple reasons suggest the association between sexual hormones and the development of meningiomas. Meningiomas are more common in females, even more for spinal meningiomas than intracranial meningiomas, and are positively correlated with breast cancer (29, 30). Most meningiomas express progesterone and somatostatin receptors (31). Spinal meningiomas expressed more androgen receptors (AR+) and estrogen receptors (ER+) than intracranial meningiomas (30).

Many studies have investigated the impact of sex hormone medication, such as oral contraception or hormonal replacement therapy (HRT), on meningiomas development. Results were inconclusive still recently (32–34). A dose-dependent relationship between the incidence and growth of meningiomas and hormonal treatment with progestin cyproterone acetate (CPA) has recently been established (35). A similar but lower risk of meningiomas has been recently reported with the use of chlormadinone acetate and nomegestrol acetate as progestin treatments (35).

Concerning HRT in menopausal patients, evidence from epidemiological studies seem to favor an increased risk of meningiomas in treated patients although a recent study failed to show an increased growth of meningiomas in HRT treated *vs.* nontreated patients (36). Until larger studies are available, it seems wise to recommend avoiding HRT in patients with meningiomas (37).

Based on studies demonstrating the expression of hormonal receptors in meningiomas, therapies targeting these receptors have been tried but have failed to show an overall favorable clinical outcome in meningioma treatment (37).

To the best of our knowledge, there are no published data on spinal meningiomas in pregnant women. However, data arising from intracranial meningiomas research suggest that the latter may enlarge during pregnancy, as noted by Cushing and Eisenhardt (38). But the rarity of this condition does not allow to clearly define the risk and the need for surgery during pregnancy. Usually, urgent neurosurgery can be indicated during pregnancy in case of neurological symptoms such as motor deficit or visual impairment (39, 40). For asymptomatic meningiomas, a multidisciplinary approach is always useful to better evaluate the pros and cons of surgery during pregnancy and following management both for maternal and fetal health, the aim being, as far as possible, to organize the surgery away from childbirth (41).

## Neuroimaging

### Magnetic resonance imaging aspect

**Figure 3:**
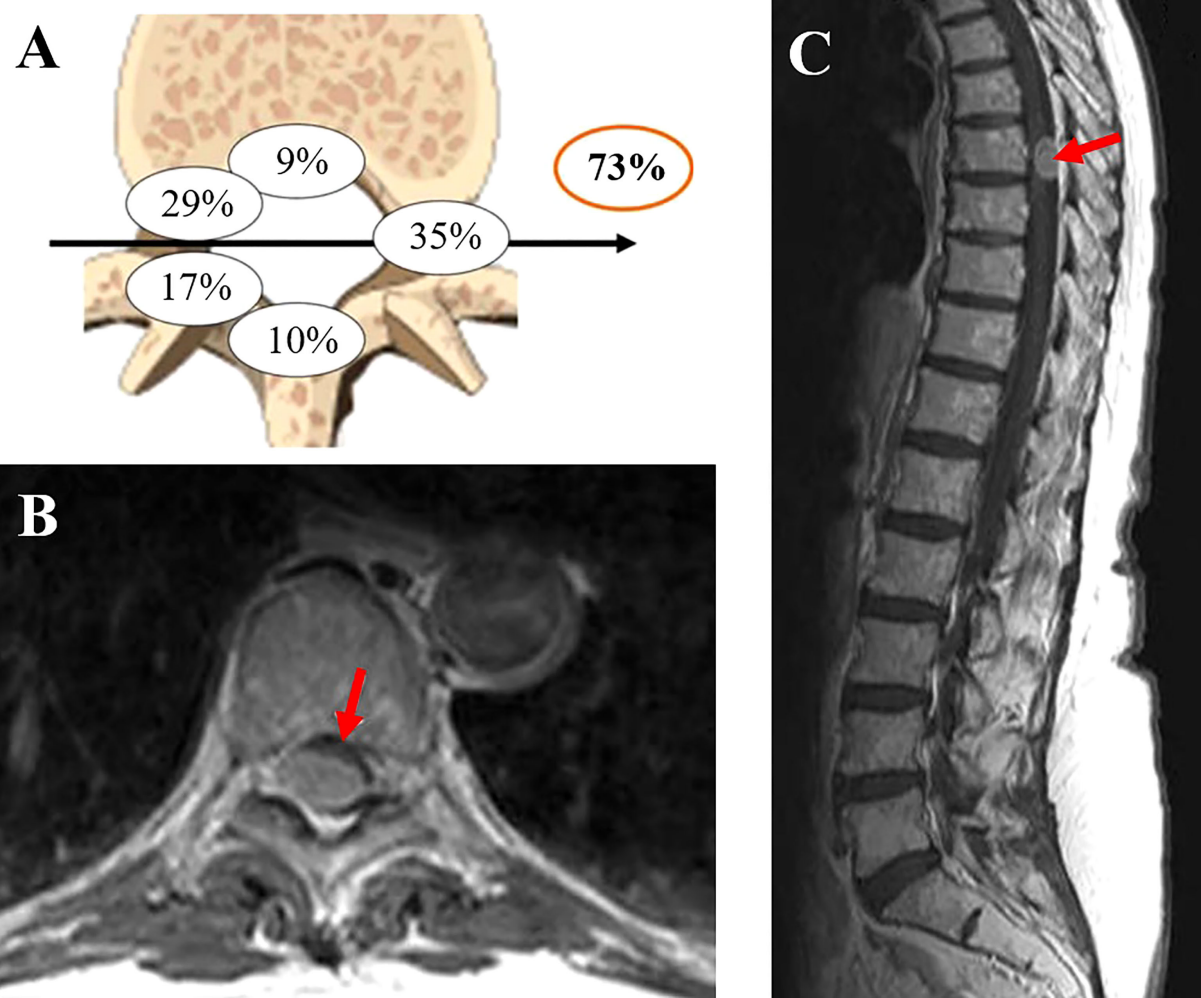
**(A)** Localization of the dural attachment (73% located ventrally to the dentate ligament). **(B, C)** Spinal meningioma aspects on MRI. The figure is adapted from reference 4 and reproduced with permission from SNCSC.

Magnetic resonance imaging (MRI) is currently the preferred diagnostic modality of intradural spinal tumors (**Figure 3**). On MRI, most spinal meningiomas show intermediate to hypo intensity on T1-weighted images (WI) and are iso- to hyperintense on T2-WI (42, 43). In fact, on T2-WI, these tumors present with a higher signal than the spinal cord but a lower signal than the surrounding fat tissue (44). They are usually well-circumscribed, with an intermediate to strong homogeneous enhancement after gadolinium injection (45). Tumors with heterogeneous enhancement seldom present with a decreased intratumoral signal on T2-WI due to calcifications, hemorrhage, or necrosis (46).

Intratumoral calcifications are reported in less than 5% of spinal meningiomas (46) but are associated with poor functional outcomes after surgical resection (4).

### Characteristic radiological features

As previously mentioned, spinal meningiomas are most commonly found in the thoracic region, in the lateral and anterolateral aspects of the spinal cord (in front of the denticulate ligament). Although a dural tail is a characteristic feature of meningiomas, it is less frequently seen in spinal than in cranial meningiomas (47).

### Radiological classification

Yeo et al. classify spinal meningiomas into four categories depending on their MRI appearance (44): Type A includes tumors with a dural attachment (with and without dural tail) that shows homogeneous enhancement after gadolinium injection; type B consists of oval-shaped tumors with hypo intense component on T2WI; type C consists of “*en plaque*” meningioma, that grows in a diffuse sheet-like aspect along with the dura mater; and type D for the rest of spinal meningiomas that could not type A, B nor C.

### A unique presentation of “en plaque” meningioma

“*En plaque*” or type C meningioma is a subgroup of spinal meningiomas. It is frequently associated with intramedullary signal changes on T2WI (44), although myelomalacia is a rare finding in spinal meningiomas (47). Moreover, type C meningioma has a larger size at diagnosis compared to well-encapsulated tumors (44). Thus, it has an increased risk of worse surgical outcomes, with postoperative arachnoiditis and higher recurrence rates after surgical resection. This might be explained by the diffuse growth pattern of these tumors, which delays clinical manifestations (44).

## Observation

More available neuroimaging modalities as MRI scans facilitated the discovery of incidental meningiomas. These lesions represent of 0.9% to 1.0% of the general population (48). Depending on the growth of the lesions, a follow-up plan is set for every individual patient. Presently, it is agreed that annual MRI scans are recommended in meningiomas of WHO grade 1 for five years. After that period, biannual MRIs can be performed.

During pregnancy, a close follow-up is recommended in patients known to have meningiomas (40).

## Management

Unlike intracranial meningiomas, spinal meningiomas do not generally invade the pia and rarely result in spinal cord edema, compared to small intracranial meningiomas that can cause significant vasogenic edema thus becoming symptomatic (49). Spinal meningiomas tend to manifest clinically once they exhibit a direct mass effect on the neural elements (50). For incidentally discovered meningiomas, clinical and radiographical observation is essential to select the best management strategy, even in the case of a documented tumor growing on serial imaging. Several factors should be considered in asymptomatic spinal meningioma before proposing tumor resection. Those factors include the patient’s age, comorbidities, and tumor size.

### Surgery

Surgery is the gold standard therapy for spinal meningiomas (51). Pseudomeningoceles or CSF leakage are the most common complications occurring in nearly 4% of cases (52). In neurosurgical practice, spinal meningiomas location and growing patterns may affect surgical results (9). The tumor location in the canal was classified into four types depending on the location of the dural attachment of the tumor and their shape (53): dorsal, lateral and ventral; dumbbell-shaped spinal meningioma being extremely rare. Ventrally attached large tumors causing spinal cord signal changes are linked with poor functional outcomes and increased risk of spinal cord traction during surgery. Severe preoperative impairment (McCormick grades III, IV, and V) that may reflect the plasticity and a vulnerable spinal cord, is significantly related to neurological deterioration postoperatively (54). Location of the meningioma throughout the spine is also important, as lumbar location gives more freedom for gross total resection (GTR) than dorsal or cervical locations, due to the absence of the spinal cord. Thoracic location is most critical as the vascularization of the spinal cord is frail (54).

Surgical removal of dorsal or lateral lesions is usually not difficult, and complete surgical resection can be achieved in 97% of cases (13). Ventrally located are less accessible, and “*en plaque*” meningiomas may not be removed totally (9).

A posterior midline approach with laminectomy can be used in the majority of dorsal or dorsolateral spinal meningiomas. Bilateral laminectomy, one level above and below the lesion, is usually sufficient for exposure in open surgery for small lesions (6, 55). The integrity of the surrounding vertebral structures should be mandatory to preserve biochemical stability, especially in cases of multilevel laminectomy or facet join disruption (56).

Anterior or anterolateral approaches are exceptional (56, 57), and must be reserved to complex anterior spinal meningiomas, because complications are more frequent, especially for CSF leakage (58). These techniques generally require a corpectomy, preferably with a unilateral approach, which implies a complementary vertebral fixation and fusion for the preservation of biomechanical stability. In some cases of anterolateral localization, when costotransversectomy and pediculectomy are needed to safely access the spinal canal and expose the lateral and anterior parts of the tumor, unilateral instrumented fusion should be done.

Excision of spinal meningiomas follows the Simpson grading (**Table 3**) regarding the totality of the resection and the treatment of the dural tail attachment (59). One of the most critical surgical steps is to define a dissection plane between the lesion and the neural elements. To access to the anterior compartment, it might be required to separate the dentate ligaments during debulking to ensure a convenient area of manipulation of the spinal cord (7). Tumor excision is accomplished using micro scissors, rongeurs, bipolar cauterization and/or ultrasonic cavitation aspirator (7, 55). Hemostasis of the epidural plexus can be achieved using surgical compression, low-intensity electrocoagulation, or hemostatic agents (55). Dural attachment resection with a patch graft suturing must be systematically performed to limit postoperative complications such as pseudomeningoceles or CSF leakage (52, 60). Finally, Kaplan-Meier survival curves showed that there is no significant difference between Simpson grade I and II, grade II GTR is more convenient in difficult surgical cases where access is difficult and risky (54).

**Table 3:** Simpson grading in meningiomas.

Grade I	GTR of the tumor as well as its dural attachments and the underlying abnormal bone
Grade II	GTR of the tumor with coagulation of the dural attachments
Grade III	GTR of the tumor without resection or coagulation of the dural attachments
Grade IV	Subtotal resection of the tumor
Grade V	Simple decompression with or without biopsy

GTR, Gross total resection.

### Minimally-invasive techniques

In 2006, Tredway et al. reported operative success using a mini-invasive hemilaminectomy technique with a tubular retractor system (55), allowing to less manipulation of the spinal cord, thus maintaining vertebral stability, decreasing blood loss and reducing hospitalization period. Since then, numerous reports have confirmed the feasibility and safety of MIS for spinal meningiomas. However, this type of approach can only be used for small lesions limited to maximum two vertebral levels (55), and must be offered to elderly and fragile patients when possible (61). However, there is still not enough evidence to recommend MIS techniques over the classical open surgery (62), because the studies advocating for MIS had small sample sizes and mixed extra- and intra-medullary tumors, resulting in confounding biases. More inspections are needed in the near future to establish specified criteria and indications for the proper usage of MIS in the treatment of spinal meningiomas.

## Intraoperative monitoring

Spinal surgery has little margin for error. Therefore, all means must be deployed to preserve the functioning of the spinal pathways and reduce the postsurgical neurological deficit. For this purpose, intraoperative neurophysiological monitoring was first introduced in 1975 by Tamaki and Yamane and has been widely used by spinal surgeons to provide information regarding the extent of tissue manipulation, tissue resection, and preservation of spinal tracts function (2, 56).

Motor evoked potentials (MEP) and somatosensory evoked potentials (SEP) are the two main neuromonitoring tools used during surgery. MEP explore the integrity of the pyramidal tracts and are primarily used in the context of anterior and anterolateral spinal lesions. SEP examine the dorsal columns and are thus mainly used in the case of posterior and posterolateral spinal lesions. The two modalities are frequently combined and offer quick and valuable surgical feedback. Intraoperative SEP and/or MEP deterioration prompt rapid surgical intervention and prevent irreversible sensory and motor pathways damage.

MEP requires stimulation of the pyramidal tracts and response recording through surface electrodes placed over a muscle of interest (for instance, intrinsic hand muscles or tibialis anterior muscle) (63–66). Direct spinal stimulation could elicit muscle responses and has been used by several groups worldwide. However, it is now known that the obtained responses could not accurately reflect the functioning and integrity of central motor pathways. Although these motor responses translate the firing of lower motor neurons, they result from the activation of various spinal tracts, including antidromic activation of dorsal column axons that have collaterals with lower motor neurons (67). Therefore, brain stimulation is recommended to monitor the integrity of corticospinal (pyramidal) tracts (64).

Brain activation could be obtained by applying magnetic or electric stimulation to the scalp. For intraoperative monitoring, electric currents are by far more practical than the magnetic field. They are delivered to the brain through “corkscrew” needles, straight needles, or surface electrodes. The latter consists of electroencephalographic (EEG) cups that could be securely fixed on the scalp (before surgery) using collodium. Anodal stimulation was more efficient than cathodal stimulation in evoking MEP. Stimulating electrodes are placed at specific sites over the motor cortex, such as C3, C4, C1, C2, Cz - 1 cm, and Cz + 6 cm (according to the 10-20 international system of electrode placement). Several montages have been proposed, like hemispheric, interhemispheric, and midline (for review, please refer to 64); each has its advantages and inconveniences that fall outside the scope of this paper and will not be discussed here.

As for SEP, this technique requires stimulation of a peripheral nerve of the upper or lower limb and response recording through electrodes positioned over the primary sensory cortex (65). Recording electrodes consist of either EEG cup electrodes fixed to the scalp using collodium or subcutaneous needle electrodes. The latter provide rapid positioning but could increase the risk of local infection or subcutaneous hemorrhage.

Concerning stimulation, median and posterior tibial nerves are usually used; other peripheral nerves can also be stimulated in certain circumstances; for instance, the ulnar nerve is chosen in lower cervical interventions. The stimuli are rectangular, of 0.2 to 0.3 ms duration, and supramaximal intensity. The latter corresponds to two times the motor threshold or three times the sensory threshold (65).

For both techniques, qualified and experimented personnel is needed to accurately detect any changes in SEP or MEP response, distinguish it from any confounder, and warn the surgical team.

## Metastatic spinal meningiomas

The literature regarding spinal meningiomas metastasis is lacking. However, according to data arising from intracranial meningiomas research, extracranial meningioma metastases (EMM) occur in 0.1% of intracranial meningiomas mainly encountered in atypical and anaplastic lesions. Most common sites of metastasis are the lungs and pleura but intraspinal and vertebral EMM also occur in a lesser percentage and are poorly described in the literature. There is no standard treatment protocol for EMM although their presence might worsen the prognosis of the concerned patients (68). EMM occurrence is independent of WHO grading and can be even present before tumor recurrence.

## Tumor recurrence

As stated previously, spinal meningiomas are benign tumors with a low recurrence rate independently of the histological grade of the cancer. In general, the recurrence rate in spinal meningiomas tend to be less than in intracranial meningiomas. The recurrence/progression of meningiomas after ten years can reach 13% (2, 69). The incidence was lower for convexity lesions (3%) than parasagittal (18%) and sphenoid ridge (34%) meningiomas. Multiple demographic, clinical and radiological factors have been associated with increased recurrence rates, such as patient age (recurrence rate higher in young patients (below 50)), tumor location (cervical), infiltrating meningioma, *En plaque* growth, extradural extension, arachnoid scarring, and partial resection (Simpson IV-V grades). Moreover, a recent series reported by Park et al. found that foraminal and thoracic location is associated with a higher recurrence rate (70).

In addition, histological types seem to influence the recurrence risk; for instance, spinal clear cell meningioma was found to have a greater recurrence rate (10).

The importance of dural attachment resection is controversial and contrasting results have been described. For instance, Nakamura et al. found that the recurrence rate was lower for Simpson Grade I than for Simpson Grade II resection (53). In contrast, a low recurrence rate can be found even without dural resection (71). In the same perspective, the add-on value of including the dural tail in the field of radiation therapy is still undetermined. Some data come from the domain of intracranial meningioma, where the radiation of the dural attachment was not found to be beneficial (*i.e.*, it did not reduce the recurrence rate) in a large retrospective series recently reported by Piper etal. (72). A better understanding of the dural tail (or dural attachment) pathophysiology is needed to guide and improve the management of this cancer.

## Functional outcomes and complications

Spinal meningiomas are frequently associated with a favorable neurological and functional prognosis (4, 73, 74).

The patients who underwent surgical decompression experienced significant post-operative improvements in Patient Reported Outcomes as measured by the Brief Pain Index and MD Anderson Symptom Inventory (75). However, anterior or calcified lesions, recurrences, cases in which the arachnoid layer has been violated, tumors invading the spinal cord and/or the vascular structures do not have the same favorable outcome (74). Therefore, the preoperative functional and neurological evaluation using the Frankel and the McCormick scales should be carried out before and after surgery (**Tables 4**, **5**). Complications are present due to neurological worsening that can be caused by the surgery itself. This includes spinal epidural hematoma, CSF leakage with or without deep or superficial infections, syringomyelia, and iatrogenic instability (4, 73, 76). In the event of early postoperative neurological deterioration, epidural hematoma should be suspected, and an emergent MRI obtained, in order to rule out this complication, the other possibility being spinal cord ischemia or edema. Epidural hematoma should be evacuated in an urgent manner to obtain rapid and total neurological recovery. In the absence of epidural hematoma and the presence of spinal cord edema, high-dose intravenous steroids should be administered. There are no exact percentages in the literature for these postoperative complications, but all publications agree on their rarity and (*i.e.*, below 5% of cases). CSF leakage is treated by lumbar drainage for 4-5 days and usually resolves without surgical revision. Superficial and deep infections are rare and must be treated with antibiotic therapy and surgical revision when required. Syringomyelia is a late onset complication. If chronic neurological impairment is attributed to the development of the evolving syrinx, subarachnoidal shunting of the cavity through an intracystic catheter should be considered. This complication is usually of bad prognosis. Iatrogenic instability should be considered mainly when back pain remains an issue with the appearance of local kyphosis on control imaging. Instrumented fusion should be considered. It could be done through a minimally invasive navigated percutaneous approach.

**Table 4:** Frankel scale evaluating functional outcomes in spinal cord injuries.

A	Complete neurological injury - no motor or sensory function below the level of the injury
B	Preserved sensation only - no motor function below the level of the injury
C	Preserved motor non-functional - some motor function observed below the level of the injury
D	Preserved motor function - useful motor function below the level of the injury
E	Normal motor - no clinically detected abnormality in motor or sensory function with normal sphincter function; abnormal reflexes and subjective sensory abnormalities may be present

**Table 5:** McCormick scale evaluating neurological outcomes in spinal cord injuries.

1	Intact neurologically, normal ambulation, and minimal dysesthesia
2	Mild motor or sensory deficit and functional independence
3	Moderate deficit, limitation of function, and independent without external aid
4	Severe motor or sensory deficit, limited function, and dependent
5	Paraplegia or quadriplegia, even without flickering movement

## Adjuvant therapy

Surgery remains the primary treatment modality for spinal meningiomas, although radiotherapy may be used as an adjuvant treatment in some cases.

Postoperative radiotherapy role is not well understood yet (14). Radiotherapy and radiosurgery are the two best substitutes for surgery in specific situations described in the literature (77–79).

Both therapeutic strategies are depicted in **Table 6**. Patients with WHO grade 1 meningiomas do not necessarily need adjuvant treatment after resection that might be sufficient for short-term tumor control (80).

**Table 6:** Indication for adjuvant treatment in spinal meningiomas.

Radiotherapy	WHO grade 3 meningioma
	WHO grade 2 meningioma with recurrence
	Subtotal resection
Radiosurgery	Elderly patients who cannot tolerate surgery
	Patients with recurrent tumors without spinal cord compression and who are not candidates for surgery

WHO, World Health Organization.

Upfront radiosurgery is usually not an option, given that small, non-compressive lesions usually require a close observation strategy, and large symptomatic lesions should undergo surgery. It may be used in fragile patients who cannot be operated. In addition, a 2-3 mm margin between the meningioma and the spinal cord is required for an effective tumoricidal dose (81). Thus, stereotactic body radiation therapy (SBRT) can be proposed for operated patients within a safe margin. The definitive SBRT dose consisted of delivering 21 Gray in three fractions. Five-year control rates with stereotactic therapy for meningiomas, varied between 70% and 100% (82). Adjuvant therapy can also be considered in WHO grade 3 meningiomas, given that these lesions are more aggressive and have a higher recurrence rate (83). Image-modulated radiation therapy (IMRT) and conventional fractionated radiation therapy have also been used to treat spinal meningioma, but SBRT is the preferred modality (77).

Chemotherapy is not used in the management of spinal meningiomas. In invasive atypical meningiomas (WHO grade 3), multiple agents have been used, including hydroxyurea, interferon α-2B, long-acting Sandostatin, and even multidrug sarcoma protocols (84). Chemotherapy can also have a role as a salvage therapy in cases of highly aggressive tumors.

## Specific situation: Spinal meingioma in pediatric population

When a spinal meningioma is diagnosed in children, a strictly follow-up must be adopted because of the high risk of developing other tumors, particularly in the context of NF2 (52). Complete surgical resection is the primary treatment modality of spinal meningiomas (85). Adjuvant radiotherapy should be recommended only for children with recurrence (85).

## Psycho-oncological aspects

Neuropsychiatric symptoms could be observed in oncology wards, including neuro-oncology. They could result either from the direct effect of the tumoral processes affecting the CNS (such in the case of intracranial meningiomas) and/or secondary to the stressful events or the adjustment processes that arise from the announcement of potentially life-threatening conditions and the related workup, surgical interventions, prognosis, and follow-up (86–88). Although these manifestations might affect patients with meningiomas, as in those with other tumors, they remain overlooked and sometimes forgotten. The majority of the few available studies published on this matter focused on intracranial meningiomas and reported the frequent occurrence of fatigue, anxiety, and depression symptoms (89, 90), as well as the lack of effect of pharmacological therapies (Methylphenidate or Modafinil) on these symptoms, compared to placebo in the randomized clinical trials that recruited patients with primary brain tumors including meningiomas (91, 92).

Fewer data are available on this matter in spinal meningiomas and involve anxiety, depression, and quality of life. For instance, in one study that considered patients with intradural extramedullary spinal tumors (of which 31.8% were meningiomas), some (50%) or extreme (14.3%) problems with anxiety and depression according to a quality-of-life questionnaire (EQ-5D-3L) were reported before the surgical intervention; the rates started to decrease from less than 1-month following the surgery to 3-12 months later, but they increased back to baseline values after one-year follow-up (93). Moreover, in another study involving patients with intradural extramedullary spinal tumors, of which 4.2% had meningiomas, 22.9% met the diagnostic and statistical manual of mental disorder criteria (DSM IV-TR) of a psychiatric disorder. In comparison, 37.5% and 12.5% of patients had mild and moderate depression symptoms according to Beck Depression Inventory, respectively (94). Furthermore, in a third study that addressed the previous limitation by including a homogeneous cohort of patients with spinal meningiomas (n=84), some or extreme problems with anxiety and depression were reported by 31% and 3.6% of patients, respectively, according to a quality-of-life questionnaire (EQ-5D-3L; 95). Here, no significant differences in problems related to anxiety and depression were found in gender or neurological status.

These facts warrant more research to further understand this clinical population’s affective and cognitive outcomes. A thorough investigation of these variables would help to (a) understand such outcomes (depression and anxiety symptoms, fear of recurrence, fatigue, coping strategies) and their clinical predictors, (b) subsequently develop specific screening tools to quantify the symptoms and identify patients at risk, and (c) implement psycho-oncological interventions that could be ideally offered in a patient-tailored manner (psychoeducation, psychosocial support, pharmacotherapeutics or psychotherapies if justified) (96). Lastly, informal caregivers of patients with spinal meningiomas seem to be still forgotten (97), and future studies are needed to evaluate and help these “hidden patients” (98), which might contribute to its turn in improving patients’ outcomes.

## Conclusion

Spinal meningiomas are intradural, slow-growing tumors, classified as a WHO grade 1 lesion in more than 70% of the cases. There is no histological difference between spinal and intracranial meningiomas.

Since intradural spinal tumors are not frequent, multicenter studies are required to fully understand and materialize the promise of targeted genetic therapies that will widen the treatment options in the future with a better clinical decision-making.

Observation should be implemented when suitable. Surgical treatment is the gold standard solution, with a principal goal of a GTR (Simpson grade I). If this GTR cannot be achieved, a SIMPSON II is advised, if possible, given the low recurrence rates. In cases of the ventral tumor where GTR is hardly achievable, small amounts of the tumor should be left. If interval growth is seen, the residual tumor can be observed and/or treated with adjuvant therapy, mainly SBRT.

Advances in surgical approaches, especially endoscopic and minimally invasive techniques, are still ongoing to minimize the risks with a better postoperative prognosis with a complete resection as a primary treatment modality.

Psycho-oncological interventions might be beneficial in patients presenting with spinal meningiomas. More research is needed to optimize screening and support patients and their informal caregivers.

## Author contributions

SA and GL: Conceptualization and methodology. NS, IL, CA, BT, FN, JF, JM, MC, RB-N and YA: Data analysis, writing-original draft preparation. MC and SA: Review and editing. SA and GL: supervision. All authors contributed to the article and approved the submitted version.
